# Lewis Base‐assisted Arylation of Unsaturated Carbonyls

**DOI:** 10.1002/chem.202302490

**Published:** 2023-10-24

**Authors:** Miran Lemmerer, Nuno Maulide

**Affiliations:** ^1^ Faculty of Chemistry Institute of Organic Chemistry University of Vienna Währinger Str. 38 1090 Vienna Austria

**Keywords:** aromatic substitution, C−C coupling, Lewis bases, organocatalysis, rearrangement

## Abstract

The combination of Lewis bases with α,β‐unsaturated carbonyls allows the in‐situ generation of enolates without the need for strong Brønsted bases. Recently developed synthetic methods employ this approach for arylation followed by elimination of the Lewis base, regenerating the alkene. This strategy has been deployed for formal α‐ or β‐C−H arylation in different contexts, namely (a) transition metal catalysis, (b) rearrangement reactions utilizing hypervalent main group elements and (c) organocatalysis. This concept article provides an overview of the developed strategies, highlighting and contextualizing their features.

## Graphical abstract

**Triple A**: Addition‐Assisted Arylation methods which make use of Lewis base‐generated enolates are described in this concept. Different fields of synthetic organic chemistry are covered, including transition metal catalysis, hypervalent main group element‐centered rearrangements and organocatalyzed aryl‐migration reactions. The conceptual similarities and differences are briefly discussed with the goal to apply this strategy in future method development.

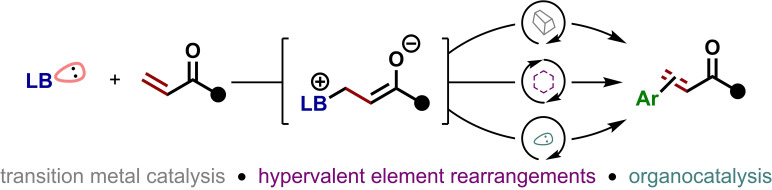

## 1 Introduction

**Scheme 1:**
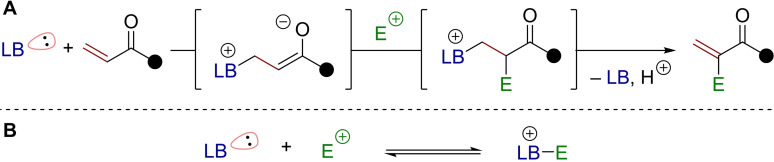
A) Lewis base‐induced enolate formation and its reaction with an electrophile. B) Necessary reversibility of Lewis base‐electrophile adduct formation. LB=Lewis base.

The development of Lewis bases as reaction mediators in organic chemistry is an ever‐growing endeavor. With their free electron pair, Lewis bases can enable the reversible formation of new bonds, allowing the transient generation of reactive intermediates during synthetic processes.^[1]^ Regardless of their specific structure, these species invariably possess increased nucleophilicity (or electrophilicity) compared to their respective starting materials. In particular, α,β‐unsaturated carbonyls can be transformed into enolate‐type intermediates in situ, which can further react through nucleophilic pathways (Scheme 1A). Once a new bond to an electrophile has been formed, elimination can occur, regenerating both the Lewis base and the initial C=C double bond. In such sequences, the reversibility of the undesired background “Lewis base‐electrophile” aggregation is crucial, as this side reaction would otherwise halt the overall process (Scheme 1B).

A by now well‐understood reaction family using carbonyls as electrophiles (“E” in Scheme 1) revolves around such a mechanism. Indeed, both the Morita−Baylis−Hillman^[2]^ and its vinylogous congener, the Rauhut−Currier reaction,^[3]^ make use of Lewis bases to promote the coupling of electron‐deficient alkenes with aldehydes and their (nitrogen)‐derivatives, enones or acrylates.^[4]^ A cornucopia of bases, including amines and phosphines but also carbenes, sulfides, and even halides have been demonstrated to catalyze these reactions in combination with Lewis acids.^[5]^ The reaction mechanism has been studied extensively, also emphasizing the influence of solvents, in particular during the elimination step.^[6]^ If coupling to a different electrophile is desired, a Rauhut−Currier dimerization must be outcompeted.

**Scheme 2:**
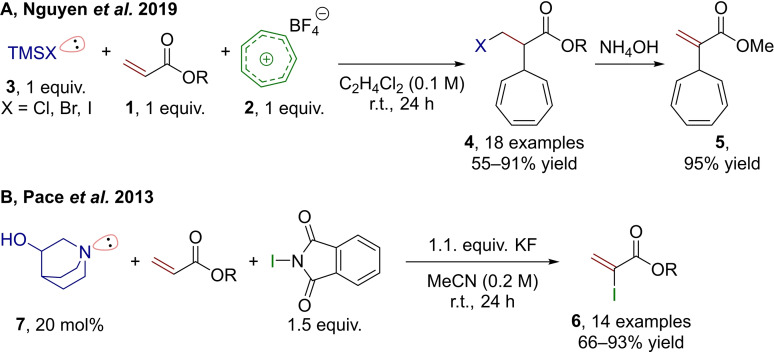
Selected examples for Lewis base‐assisted, non‐Morita−Baylis−Hillman α‐functionalization reactions of unsaturated carbonyls. TMS=trimethylsilyl.

Recent developments have gone beyond the combination of electron‐deficient alkenes and carbonyls, and ventured into other types of C−C coupling reactions. Nguyen et al., for example, have disclosed a direct reaction between acrylates **1** and tropylium halides (directly or reversibly generated in situ from **2** and a trimethylsilyl halide (**3**)), in which the halide assumes the role of the Lewis base and the tropylium counterion is the electrophile (**4**, Scheme 2A).^[7]^ The formal Lewis base‐electrophile adduct formation is a simple collapse of the tropylium salt and therefore reversible, rendering it irrelevant for the outcome of the reaction. Simple treatment with aqueous ammonia regenerates the double bond (**5**). In contrast to this β‐halogenation method with the possibility for subsequential elimination, α‐iodination forming vinyl iodides (**6**) directly, can be achieved via a simple protocol using the amine 3‐quinuclidinol (**7**) as the mediator, *N*‐iodophthalimide as the electrophile and KF as an additional base (Scheme 2B).^[8]^ Again, the adduct‐forming side reaction in this case is not detrimental, as it itself produces a competent I^+^‐source.

This concept article will focus on new approaches towards Lewis base‐assisted (“Morita−Baylis−Hillman‐style”) arylation reactions (formal Csp^2^−Csp^2^ coupling), which creatively harness Lewis base‐induced enolate formation.

## 2 Arylation Reactions

**Scheme 3:**
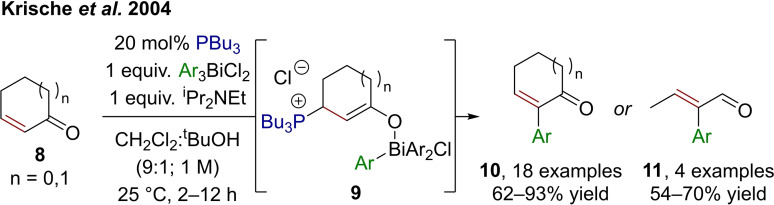
A phosphine‐catalyzed arylation method using an aryl bismuth(V) complex as the electrophile. Ar=aromatic substituent.

As early as 2004, Krische et al. reported a method for the α‐arylation of cyclic enones (**8**) and–exclusively (*E*)‐configured–crotonaldehyde using an aryl bismuth(V) complex (Scheme 3).^[9]^ Therein, the strong Lewis base tributyl phosphine adds to the α,β‐unsaturated carbonyl to generate an enolate which, in turn, is a competent ligand for bismuth and can exchange with chloride. This preassembly allows **9** to collapse, forming the C^α^‐aryl bond by release of Ar_2_BiCl. After elimination of tributylphosphine and HCl, the desired product **10** or **11** is formed. Key for a smooth transformation is the compatibility of the phosphine with the initial bismuth(V) complex (as described in Scheme 1), as well as the solvent mixture, which presumably allows for a low energy barrier in the elimination step. Beyond the reported enones and enals, the authors mention that other pronucleophiles lacking β‐substitution engage in anionic polymerization rather than the desired α‐arylation.

**Scheme 4:**
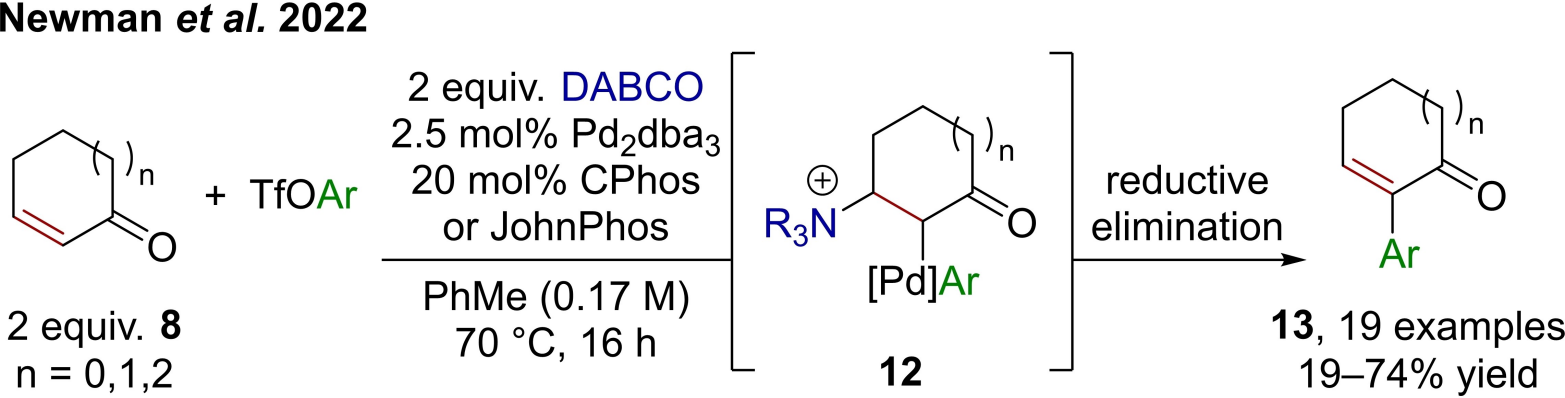
A palladium‐catalyzed coupling of DABCO‐generated enolates to aryl triflates. CPhos=2‐dicyclohexylphosphino‐2’,6’‐bis(*N*,*N*‐dimethylamino)biphenyl, JohnPhos=(2‐biphenyl)‐di‐*tert*‐butylphosphine.

While this process relies on a stoichiometric aryl‐metal component, a recent catalytic method using an in‐situ generated arylpalladium complex as the electrophile was disclosed by Newman et al. (Scheme 4).^[10]^ Cyclic enones (**8**) are thus C−C coupled in α‐position to aryl triflates, with the assistance of the Lewis base DABCO (1,4‐diazabicyclo[2.2.2]octane). Remarkably, this method is regiocomplementary to traditional, palladium‐catalyzed Heck‐type transformations, which would typically lead to β‐arylation.^[11]^ The authors studied the reaction mechanism through deuterium labeling, using competing Morita−Baylis−Hillman reactions and by probing the electronic requirements. These studies led to the conclusion that indeed an ammonium enolate must be generated, which then reacts with the arylpalladium complex, forming first a Pd−O bond followed by a metallotropic shift to C‐palladated **12**. Reductive elimination regenerates Pd(0) and liberates product **13** following DABCO liberation. While this mechanism would, in principle, allow for both palladium and Lewis base to be used in catalytic amounts, optimization showed the need for two equivalents of DABCO to achieve high yields, since it also plays the role of a Brønsted base.

**Scheme 5:**
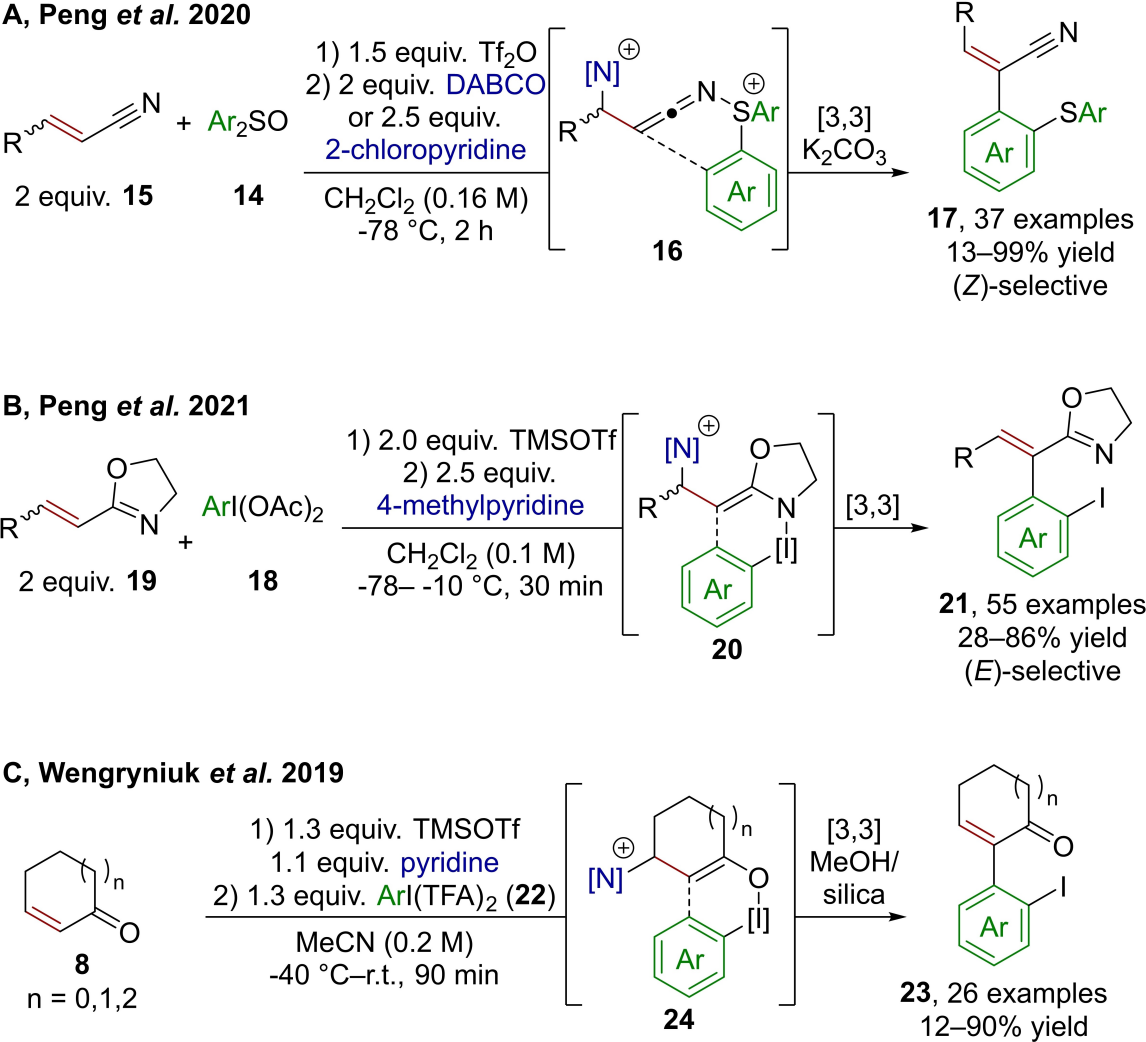
Arylation of nitriles, oxazoline and ketones through [3,3]‐sigmatropic rearrangements using highly electrophilic, in situ generated, S(IV) and I(III) species. TFA=trifluoroacetate.

Rearrangements are a powerful synthetic tool to forge Csp^2^−Csp^3^ bonds without the requirement for a mediating transition metal. Using the redox‐active main group elements sulfur and iodine, Peng et al. reported the arylation of specific electron‐deficient alkenes via [3,3]‐sigmatropic rearrangements.[^12^, ^13^] Leveraging electrophilic activation of aryl sulfoxides (**14**) with triflic anhydride, addition of α,β‐unsaturated nitriles (**15**) is enabled, leading to the assembly of complexes prone to attack by a subsequentially added nitrogen Lewis base (DABCO or 2‐chloropyridine), forming ketenimine **16** in situ (Scheme 5A). Upon formation of **16**, a [3,3]‐sigmatropic rearrangement forms the desired new C−C bond and rearomatization follows.^[12]^ Elimination of the Lewis base is ultimately induced by K_2_CO_3_. The premixing of **14**, **15** and trifilic anhydride prior to base addition is crucial to achieve high yields. Remarkably, this sequence leads selectively to (*Z*)‐configured products (**17**), with quantum chemical calculations suggesting a significantly higher transition state energy for the elimination to the (*E*)‐configured product.

This selectivity stands out even more when compared to a similar process developed shortly after and utilizing I(III) (**18**) instead of S(IV) in combination with vinylic oxazoles **19** (Scheme 5B).^[13]^ Therein, electrophilic activation is achieved using TMSOTf and a rearrangement (**20**) conceptually similar to that shown in Scheme 5A leads to *o*‐iodo vinyl arenes **21**, exclusively with *E*‐configuration again attributed to a selective E1cB elimination step, as supported by quantum chemical computations.

As discussed before, cyclic enones (**8**) are generally prone to Lewis base attack due to their pronounced electrophilicity. This enabled the use of I(III) reagents (**22**) to achieve α‐arylation (**23**), as disclosed by Wengryniuk et al. in 2019 (Scheme 5C).^[14]^ In this work, activation of the enone with TMSOTf and pyridine forms a pyridinium silyl enol ether which attacks the electrophilic iodonium trifluoroacetate species (**24**). When the reaction is performed using crotonaldehyde, a mixture of alkene isomers is obtained.

**Scheme 6:**
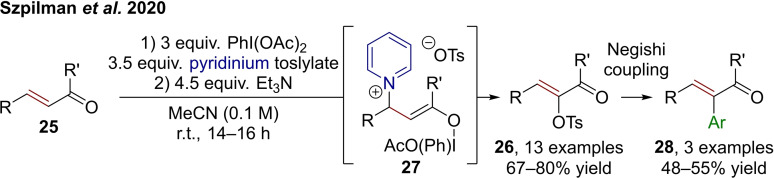
Umpolung of α,β‐unsaturated ketones delivering α‐tosyloxy products **26** and the possibility to access **28** through metal‐catalyzed processes.

While delivering similar products, these iodine(III)‐centered reactions are in stark mechanistic contrast to cationic aryl migration reactions induced by I(III)‐mediated α‐umpolung.^[15]^ However, a combination of such an umpolung strategy of α,β‐unsaturated ketones with pyridine as the Lewis base allows for the formal α‐oxotosylation of enones (**25**), as demonstrated by Szpilman et al. (Scheme 6).^[16]^ (Diacetoxyiodo)benzene is used as an oxidant while pyridinium tosylate plays a triple role as Brønsted acid,^[17]^ Lewis base donor, and nucleophile to deliver the umpoled products **26** via intermediates **27** (electrophilic on the initial α‐carbon). While this process does not directly deliver α‐arylated products **28**, a subsequent Negishi coupling can be conducted to that end (cf. Scheme 6).

**Scheme 7:**
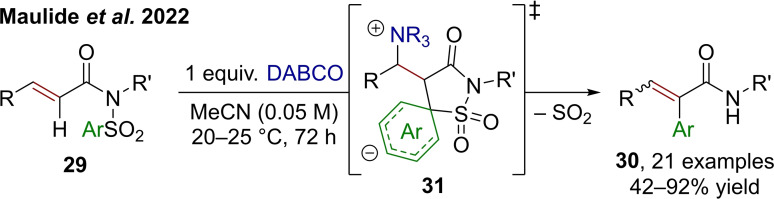
Synthesis of α‐aryl acrylamides via Smiles rearrangement mediated by DABCO.

Aryl migration reactions can progress through a range of conceptually different mechanisms. As discussed above, sequences relying on [3,3]‐sigmatropic reactions deliver *ortho*‐functionalized products. In contrast, a different approach, often termed Smiles‐rearrangement, proceeds through *ipso*‐substitution of tethered aromatic (or vinyl) groups.^[18]^ Over the last decade, a plethora of reactions utilizing such a rearrangement has emerged, enabling the synthesis of saturated α‐aryl carboxamides starting from *N*‐sulfonyl carboximides.^[19]^ While these methods usually rely on radical sequences, Maulide et al. have recently approached these Smiles cascades from a different, polar, point of view—ultimately exchanging arene and hydrogen under loss of SO_2_ (Scheme 7).^[20]^ Using the Lewis base DABCO, an aryl migration on α,β‐unsaturated *N*‐arylsulfonyl carboximides **29** is induced to afford a range of α‐aryl acrylamides **30**. It is worthy of note that the ionic nature of this process, proceeding through a spirocyclic (Meisenheimer‐)transition state (**31**), requires the aromatic group to carry electron‐withdrawing substituents. Interestingly, the pivotal Lewis base plays several roles in the reaction mechanism, as unearthed through computational analysis. Enolate formation, proton transfer during the elimination step, and liberation of SO_2_ are all either induced or assisted by DABCO.

**Scheme 8:**
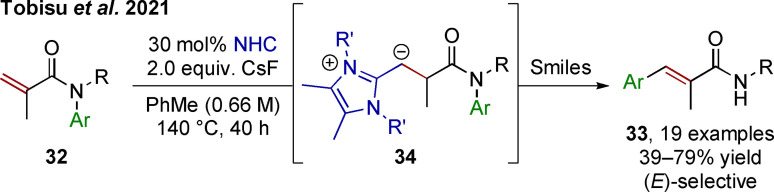
NHC‐catalyzed Aryl migration into the β‐position of methacrylamides. R′=*o*,*o*′‐dimethyl‐*p*‐methoxy phenyl.

The Smiles rearrangement can also be used beyond α‐arylation. Recent independent reports by Tobisu et al.^[21]^ and Zhou et al.^[22]^ have shown how β‐arylation of acrylamides can be achieved employing such a migration reaction (Scheme 8). Starting from *N*‐aryl methacrylamides **32**, a variety of cinnamides **33** are synthesized using the power of N‐heterocyclic‐carbene (NHC) catalysis at high temperature. Conjugate addition to the alkene is followed by hydrogen shift, forming β‐anion equivalent **34** (often referred to as a deoxy‐Breslow‐ or vinylogous Breslow intermediate). *Ipso*‐attack on the aryl group initiates the anionic Smiles rearrangement which subsequentially leads to elimination, liberating selectively (*E*)‐cinnamides. This organocatalytic process is a valuable alternative to classic, palladium‐catalyzed Heck‐type reactions.

## 3 Summary and Outlook

Traditionally, reactions relying on Lewis base‐mediated enolate formation have focused on Morita−Baylis−Hillman‐type alkylations. Recent discoveries have shown, however, that this strategy can also be applied for arylation reactions in different contexts such as transition metal‐catalyzed processes, oxidative sigmatropic rearrangements, or migration reactions through *ipso*‐substitution. Over the last few years, we have seen an awakening in the synthetic community to exploit Lewis bases by developing new methods and study their intricacies. A glance into the crystal ball of organic chemistry allows us to expect further developments in the Lewis base/unsaturated carbonyl reactivity‐space.

## Conflict of interest

The authors declare no conflict of interest.

4

## Biographical Information

*Dr. Miran Lemmerer studied chemistry at the University of Vienna and graduated in 2023 under the supervision of Prof. Dr. Nuno Maulide. During his studies, he spent several months each in Lund, Sweden (Erasmus program) and Kyoto, Japan in the group of Prof. Dr. Hideki Yorimitsu (supported by DoSChem). His research focus is on the development of new methods for the preparation of organic compounds (revolving around electrophilic amide activation, iminium ions as building blocks, and ionic aryl migration reactions) with the ultimate goal to extend our understanding of chemistry. He likes ice cream*.

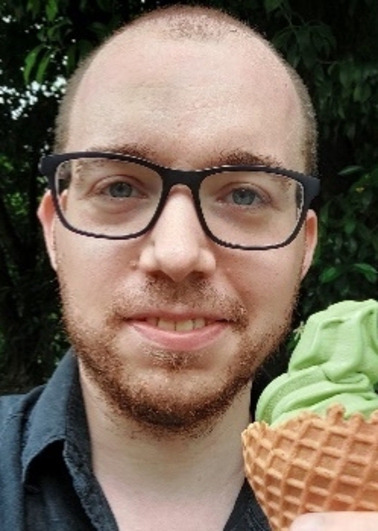

## Biographical Information

*Nuno Maulide studied at the Instituto Superior Técnico and obtained an M.Sc. degree from the Ecole Polytechnique. Following Ph.D. studies (Prof. István Markó) at the Université catholique de Louvain (2007), he moved to Prof. Barry Trost's group (Stanford University), before becoming Max‐Planck Research Group Leader (MPI für Kohlenfoschung) in 2009. In 2013, he moved to the University of Vienna, where he is Full Professor of Organic Synthesis and Adjunct PI at the Research Center for Molecular Medicine (CeMM) of the Austrian Academy of Sciences. His research involves unconventional reactivity in organic chemistry and the development of small‐molecule tools for biochemical and medicinal applications*.

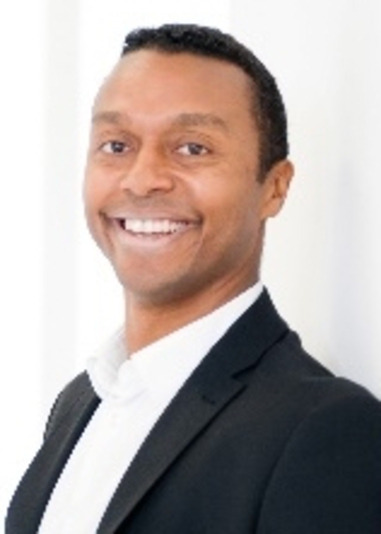

## Data Availability

The data that support the findings of this study are available from the corresponding author upon reasonable request.
